# Protest suicide: considerations for psychiatrists and psychologists

**DOI:** 10.3389/fpsyt.2023.1213420

**Published:** 2023-06-15

**Authors:** Sara Nooraeen, Paul E. Croarkin

**Affiliations:** Department of Psychiatry and Psychology, Mayo Clinic, Rochester, MN, United States

**Keywords:** suicide, protest suicide, mental health, protest, self-immolation, Iran

## Introduction

On 26 December 2022, Mohammad Moradi, a 38-year-old Iranian student living in France, died by suicide to draw global attention to Iranian protests against the ruling power. Before he threw himself into the Rhône River, Moradi shared a video and said: “when you see this, I will be dead. I don't have any mental disorders or life problems. I have a good life, but I can't be unresponsive to what is happening in Iran. I want to sacrifice myself for my country”. Besides the social and political aspects of this tragic event, his suicide highlighted the need to take a look again at suicide as an act of protest. Protest Suicide is a rarely studied social issue especially, through the lens of psychiatry and mental health. Although political issues from riots to revolutions could contribute to mental health problems such as depression and post-traumatic stress disorder (PTSD) (1), suicide is still one of the most controversial consequences. Similarly, pure medicalization of protest suicide is not possible. The sheer complexity of self-inflicted death and its confluence with human concepts, such as freedom and self-sacrifice make this subject more intricate and protest suicide has also been referred to as “altruistic suicide” (2). However, psychiatric aspects of suicide should not be ignored in the shadow of heroism or protest.

## History of protest suicide

There are numerous instances of protest suicide during past centuries (3). Most often, protest suicide occurs in developing countries (4, 5), and there are few documented instances in Western countries (6). Self-immolation, hanging, poisoning and as we see in Moradi's case, drowning are the most commonly known methods of protest suicide. There is a religious and cultural relationship between self-immolation and the society in which protest suicide happened, such as Buddhism (7). Similarly, in some cultures, especially in Asia, protest suicide through the mean of self-immolation could be accepted as an honor (8). But in Western countries, it generally is associated with mental illness and substantial stigma (9).

There are various inspiring factors for protest suicide and not all are political. protest suicide can be a means to protest against injustice in the workplace (10, 11), family and living environment (12–14). Protest suicide can be compared with some other types of protest such as hunger strikes, which can cause death (15), suicide terrorism which is the act of suicide and killing other people (16) and martyrdom. But what is essential for a protest suicide to attain the status of a heroic positive act is societal reaction that is typically shaped by media (2). Generally, those who take their life to protest against oppression and injustice, were respected by society. Those who die in this manner are often regarded as martyrs with statues built to streets named to memorialize them.

Protest suicide has a dual purpose: rebelling against oppressive authoritarianism and effecting needed and transformative change in society (17). An action-taker's expectation is to persuade the government to make some reforms and evoke a societal response and encourage them to move. So, protest suicide needs to be spread through the media to make the message of action-taker heard (18). The spread of protest suicide news often does yield changes in that society. Sahar Khodayari, also known as Blue Girl, was an Iranian football fan who set herself on fire against the rule which banned women from entering football stadiums in Iran. One month later, Iranian women were allowed to attend a football match in Iran for the first time in 40 years. Sometimes, these changes happen in a broader scope. Self-immolation of Mohamed Bouazizi, a Tunisian street vendor who burnt himself to death in 2010 against confiscation of his wares, is known to have catalyzed the Tunisian revolution and a series of protests and revolutions in some other Arab countries, referred to as the “Arab Spring”.

## Protest suicide through the lens of psychiatry

Tragically, some situations lead protesters to choose suicide as a last resort. It raises an important role of psychiatrists and mental health providers addressing societal mental health during riots and revolutions. Without minimizing the role of totalitarian authorities in making social injustice that causes protest suicide, here, we aim to discuss the negative aspects of protest suicide on individuals and societies. From a psychiatric perspective, suicide is always an emergency, irrespective of contributing factors such as the shadow of self-sacrifice. All human life is important and valuable. Protest situations and social unrest could put a significant burden on individuals' mental states (19). In this situation, suicidal ideation is not improbable. So, it is very important to suicide warning signs to be taught by mental health professionals. In these societies, all people and protestors should know the signs of suicide risk and how to treat someone who is at risk of suicide. Altruistic efforts as a group bolster social connectedness and are associated with lower rates of suicide, so cross-cultural societal awareness of protest suicide is important. Our psychiatric research community should also study protest suicide and related societal stressors more rigorously.

Glorification and idealization of protest suicide should be avoided altogether to avoid a contagion effect. Also, reporting the protest suicide news should meet criteria to prevent the “Copycat effect” or “Werther effect” which refers to the suicidal behavior that occurs after exposure to another person's suicide. Bouazizi's protest suicide resulted in more than ten imitation self-immolations in different countries. It is essential to prevent imitation suicides after a protest suicide. The importance of social media in contagion of suicide clusters is of note, since, it has possible harmful effects on people at risk of suicide. The internet, social media, and evolving digital technologies present more knowledge gaps and urgency for further research focused on protest suicide as there both contemporary risks for increased incidence of protest suicide and unique opportunities for preventions. At present, our psychiatric field is not well positioned to address these risk or opportunities for preventions.

Psychiatrists and psychologists likely have different conceptualizations of protest suicide compared to sociologists, philosophers and politicians. Even the term “Protest Suicide” is not well-known among psychiatrists and psychologists. Mental health clinicians who consider protest suicide as irrelevant and imagine that it occurs among non-mentally ill persons fail to consider diagnoses of post-traumatic and acute stress disorders. Although the sociological dimensions of suicide can heighten risk, suicide almost always has psychological underpinnings. Mental health providers have an important role in addressing, studying and preventing all types of suicides alongside other specialists. Family survivors of those who die by protest suicide experience guilt, shame and anger and are at risk of suicide themselves.

## Directions for future studies

Psychiatrists, psychologists and other mental health professionals have an important duty and role in addressing protest suicide and the advancement of related knowledge base. There are several directions for future studies from the lens of psychiatry and psychology. While understanding the social and cultural determinants of protest suicides is important in terms of prevention, clinicians should consider how the individual's psychology and temperament modulate the psychosocial stressors by either increasing risk or in suicide protective ways.

## Author contributions

Conceptualization: SN and PC. Supervision: PC. Writing: SN. All authors contributed to the article and approved the submitted version.
